# Early oral feeding versus nasojejunal early enteral nutrition in patients following pancreaticoduodenectomy: a propensity score-weighted analysis of 428 consecutive patients

**DOI:** 10.1097/JS9.0000000000000786

**Published:** 2023-09-22

**Authors:** Wei Jing, Shengyong Wu, Suizhi Gao, Xiaohan Shi, Wuchao Liu, Yiwei Ren, Liu Ouyang, Kailian Zheng, Shiwei Guo, Cheng Wu, Gang Jin

**Affiliations:** a Department of Hepatobiliary Pancreatic Surgery, Changhai Hospital Affiliated to Navy Medical University; b Department of Military Health Statistics, Navy Medical University, Shanghai, China

**Keywords:** early oral feeding, nasojejunal early enteral nutrition, pancreaticoduodenectomy, postoperative outcomes

## Abstract

**Background:**

Notwithstanding that significant medical progress has been achieved in recent years, the optimal nutritional support method following pancreaticoduodenectomy (PD) remains uncertain. This study compared the safety and feasibility of early oral feeding (EOF) with nasojejunal early enteral nutrition (NJEEN) after PD.

**Methods:**

A retrospective cohort study was conducted on 428 consecutive patients who underwent PD between August 2018 and December 2020. During the first study phase, the routine postoperative feeding strategy was NJEEN, later replaced by EOF during the second study phase. The primary outcome was the incidence of delayed gastric emptying (DGE) following PD. Propensity score weighting was used to control for confounding factors.

**Results:**

Four hundred forty patients underwent PD during the overall study period, with 438 patients aged 18 years and older. Ten patients experienced accidental tube dislodgement or migration and were excluded from the study based on the exclusion criteria. Finally, 211 patients and 217 patients underwent EOF and NJEEN, respectively. After propensity score weighting, it was observed that patients who underwent postoperative EOF experienced a significantly lower DGE (B/C) rate compared to those who underwent postoperative NJEEN [7.38% (31/424) vs. 14.97% (62/413), *P*=0.0005]. Subgroup analyses according to the presence of soft pancreatic texture yielded consistent results. The EOF group exhibited lower DGE grade, DGE (B/C) rate [5.90% (11/194) vs. 22.07% (43/193), *P*<0.0001], postoperative gastrointestinal endoscopic intervention rate, and Clavien–Dindo Grade III or higher rate.

**Conclusions:**

EOF is superior to NJEEN in reducing the incidence of grade B/C DGE after PD. The EOF procedure is safe and feasible and should be recommended as the optimal postoperative feeding method following PD.

## Introduction

Highlights

- The optimal nutritional support method after pancreaticoduodenectomy (PD) is still uncertain.
- This retrospective cohort study compared the safety and feasibility between early oral feeding (EOF) and nasojejunal early enteral nutrition after PD.
- The propensity score weighting analysis showed that patients who used nasojejunal early enteral nutrition had a significantly higher delayed gastric emptying (B/C) rate than EOF, especially in the subgroup of soft pancreas. Therefore, the EOF should be recommended following PD.

Pancreaticoduodenectomy (PD) remains the mainstay of treatment for periampullary and pancreatic head carcinomas. However, perioperative complications are common, with incidence rates ranging from 40 to 60%^1–3^. Malnutrition is widely recognized as a significant predictor of postoperative morbidity and mortality following pancreatic surgery^4^. Adequate nutrition plays a crucial role in reducing perioperative complications, such as abdominal infection, wound healing issues, and even gastroanastomotic leaks^5–7^. Although diverse nutritional support methods are currently available, the optimal approach after PD remains uncertain. In this respect, an international survey revealed a lack of consensus on postoperative nutrition management following Whipple surgery^8^.

Some systematic reviews have suggested that early enteral nutrition (EEN) is a safe and effective strategy that can significantly reduce the length of hospital stay for PD patients^9,10^. The European Society for Parenteral and Enteral Nutrition (ESPEN) recommends EEN with a needle catheter jejunostomy or nasojejunal tube (NJT) following gastrointestinal or pancreatic surgery^11^. However, a randomized multicenter controlled trial reported that nasojejunal early enteral nutrition (NJEEN) is associated with increased postoperative complications and is thus not recommended^12^. In recent years, with the increasing adoption of enhanced recovery after surgery (ERAS) protocols, more surgeons are exploring the potential of early oral feeding (EOF) after PD.

A systematic review indicated no significant differences in outcomes between oral diet, enteral nutrition (EN), and total parenteral nutrition after PD, and on-demand NJT feeding with EOF might be appropriate after PD^13^. Another randomized controlled trial demonstrated the feasibility and safety of EOF following pylorus-preserving pancreaticoduodenectomy (PPPD)^14^. Besides, the International Study Group of Pancreatic Surgery (ISGPS) recommends EOF after pancreatic surgery within ERAS protocols. However, very few studies have directly compared EOF with routine NJEEN after PD, especially in patients with soft pancreatic texture who are at higher risk of complications.

In this real-world study, we sought to compare the safety and feasibility of EOF with NJEEN after PD and specifically explore their effects in patients with soft pancreatic texture after PD.

## Materials and methods

### Patients

The retrospective cohort study included patients who underwent classic Whipple pancreaticoduodenectomy or pylorus-preserving pancreaticoduodenectomy at our centre from August 2018 to December 2020. Patients scheduled for open PD and aged older than or equal to 18 years with an American Society of Anesthesiologists (ASA) status less than 4 were eligible for the study. Patients with accidental dislodgement or migration of the nasojejunal tube were excluded. Clinical data on demographics, treatments, intraoperative details, pathology, and clinical outcomes were collected from the prospective database.

This study was carried out in accordance with the revised Declaration of Helsinki and approved by our hospital’s Ethics Committee. Besides, this study has been submitted to the Clinical Trials Register (NCT05573399). We emphasized to NJEEN patients and their families the significance of nasojejunal feeding tube placement before and after the operation in hospital to enhance adherence. Surgical patients were categorized into two groups based on the timing of surgery and the standard feeding protocol they received. The study adhered to the Strengthening the Reporting of Observational Studies in Epidemiology (STROBE) reporting guidelines for observational studies, and the findings were reported in accordance with the Strengthening The Reporting Of Cohort Studies in Surgery (STROCSS) criteria^15^. Supplemental Digital Content 2, http://links.lww.com/JS9/B85.

### NJT feeding

NJEEN was adopted as the routine postoperative feeding strategy during the first study phase (August 2018–July 2019). Jejunal feeding tubes were inserted into the jejunum during surgery to facilitate the delivery of enteral nutrition (Flocare Bengmark Naso-Intestinal tube). Additionally, the anesthesiologist inserted a nasogastric tube into the stomach, advancing it ~30 cm until the tip of the NJT entered the efferent loop following the duodenal or gastrojejunostomy. It was then securely taped between the nostrils and the earlobe. The NGT was inserted on postoperative day (POD) 1 for gastrointestinal decompression and to prevent anastomotic leakage, and it was removed on POD2 when the drainage volume was less than 300 ml per 24 h.

On POD1, a 25 ml/h infusion of 5% glucose saline was initiated in the NJEEN group via the NJT. Enteral nutrition [Enferal Nutritional Suspension (TPF) by Nutricia Pharmaceutical (Wuxi) Co.] was initiated on POD2 at a rate of 25 ml/h, with an increment of 25 ml per 24 h until the total amount reached 1500 ml per day, depending on the patient’s tolerance. The diet was administered over a 20-h period using a peristaltic pump at a steady flow rate. On POD3, patients were allowed to resume oral intake. When oral intake was deemed sufficient on POD6, the NJT was removed. However, if delayed gastric emptying (DGE) occurred before the NJT was removed, (EN continued to be administered to NJEEN patients.

### Early oral feeding

During the second study phase (July 2019–December 2020), the routine postoperative feeding strategy was EOF. The NGT was removed when the drainage volume was less than 300 ml per 24 h on POD2. Liquid diets were allowed on POD2, and solid foods were introduced on POD5.

If DGE occurred after NJT extraction, total parenteral nutrition was administered to the EOF and NJEEN groups. NJTs were placed under endoscopic guidance on POD14, and EN continued to be administered. The combination of parenteral nutrition was determined based on the patient’s medical condition.

### Surgical approach

A single surgical team performed the pancreaticoduodenectomy alongside a retroperitoneal lymphadenectomy. The PD procedure involved end-to-side, duct-to-mucosa, double-layer continuous suturing pancreatojejunostomies, hepaticojejunostomies, gastrojejunostomies, or duodenojejunostomies. A silica gel drainage tube was inserted into the pancreatic duct, and all hand-sewn anastomoses were performed.

### Covariates and outcomes

Baseline characteristics included demographic data, medical history, and surgical conditions. The primary outcome was the incidence of delayed gastric emptying following PD. Secondary outcomes included the postoperative situation, application of postoperative management, length of stay), and costs of patients. Details on covariates, and outcomes are provided in the Supplementary Appendix, Supplemental Digital Content 1, http://links.lww.com/JS9/B84.

Postoperative pancreatic fistula (POPF), PPH (postoperative hemorrhage), and DGE were diagnosed and classified according to the International Study Group of Pancreatic Surgery (ISGPS) definitions^16–18^. For the assessment of POPF, only grades B and C were considered.

### Statistical analysis

Continuous variables were presented as mean (SD) or median (interquartile range) depending on their distribution. Categorical variables were represented as the number *n* (%). Student’s *t*-test or Mann–Whitney U test was used for continuous variables, and Pearson χ^2^ or Mann–Whitney U tests were used for categorical or ordinal variables.

To address potential confounding factors, we employed a propensity score weighting (PSW) method, specifically the inverse probability of treatment weighting method, referred to as model 1. The propensity score was derived using a logistic regression model, incorporating adjustments for the listed covariates. Adequacy of weighting was assessed by examining standardized mean differences for each baseline covariate, and a value less than 0.10 indicated that the groups were adequately balanced and there was no significant disparity between them.

Subgroup analyses were conducted on patients with soft pancreatic texture, which was determined by the surgeon during operation, and PSW was used to evaluate the performance of different groups.

To assess the robustness of our findings, we conducted two sensitivity analyses using propensity score matching (Model 2) and multivariable regression analysis (Model 3) to mitigate the impact of confounding variables and reduce the likelihood of selection bias. Details on sensitivity analyses are provided in the Supplementary Appendix, Supplemental Digital Content 1, http://links.lww.com/JS9/B84.

All statistical tests were two-tailed, and a significance level of *P* less than 0.05 was used unless otherwise specified. All statistical analyses were carried out using SAS version 9.4 (SAS Institute Inc) and R version 4.0.4 (R Foundation for Statistical Computing).

## Results

**Figure 1:**
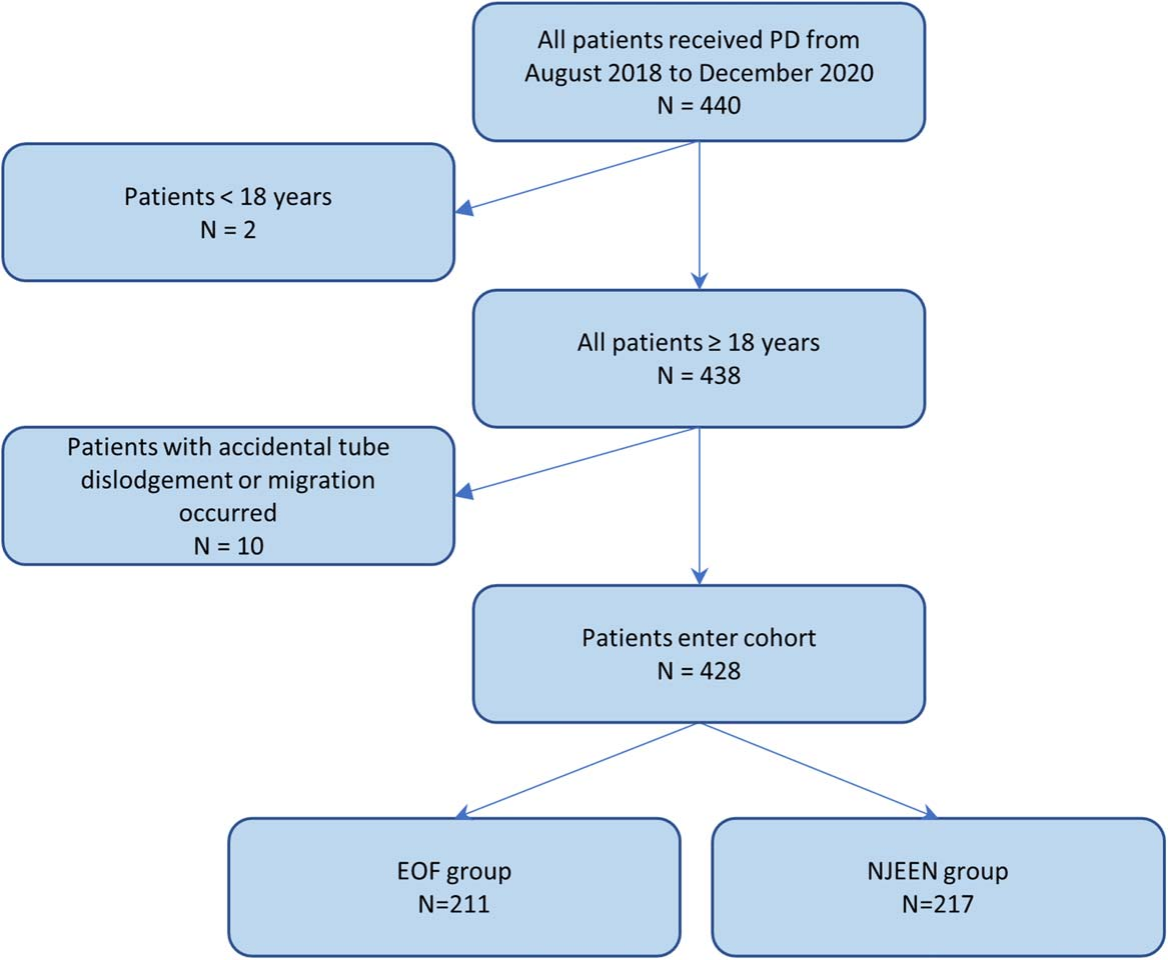
Flow diagram illustrating the selection process of the target population. EOF, early oral feeding; NJEEN, nasojejunal early enteral nutrition; PD, pancreaticoduodenectomy.

Four hundred forty patients underwent PD between August 2018 and December 2020, with 438 patients aged 18 years and older included in the study. Ten patients were excluded due to accidental tube dislodgement or migration according to the predefined exclusion criteria. Among the remaining patients (*n*=428), 211 and 217 patients underwent EOF and NJEEN, respectively (Fig. 1).

### Descriptive overview

**Figure 2:**
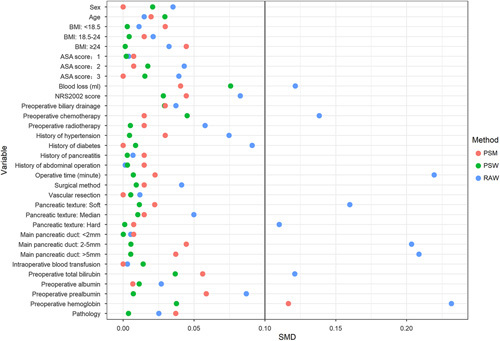
Standardized mean differences (SMD) of patients, patients after propensity score weighting, and patients after propensity score matching. ASA, American Society of Anesthesiologists; PSM, propensity score matching; PSW, propensity score weighting; RAW, raw data.

Table 1 provides the baseline characteristics for the entire cohort and the two groups. Patients that underwent EOF were associated with more blood loss, shorter operative time, lower preoperative total bilirubin and haemoglobin levels, a lower incidence of soft pancreatic texture, a larger size of the main pancreatic duct, and higher rates of NRS2002 score less than 3, preoperative radiotherapy, preoperative chemotherapy, and a history of diabetes (Table 1). After PSW, the standardized mean differences for all preoperative covariates were less than 0.10, indicating comparable weighted populations in both groups (Table 1, Fig. 2). Details of the baseline on sensitivity analyses are provided in the Supplementary Appendix, Supplemental Digital Content 1, http://links.lww.com/JS9/B84.

**Table 1:** Patient characteristics before and after propensity score matching.

		Before PS methods					After PS weighting				
Variables	Classification	All cohort (*N*=428)	EOF group (*N*=211)	NJEEN group (*N*=217)	*P*	SMD	All cohort (*N*=837)	EOF group (*N*=424)	NJEEN group (*N*=413)	*P*	SMD
Sex, *n* (%)	Male	250 (58.41)	127 (60.19)	123 (56.68)	0.4617	0.04	496 (59.28)	247 (58.26)	249 (60.33)	0.5416	0.02
	Female	178 (41.59)	84 (39.81)	94 (43.32)			341 (40.72)	177 (41.74)	164 (39.67)		
Age, mean±SD		60.85±11.31	60.77±11.33	60.94±11.31	0.8783	0.01	61.25±15.57	61.08±15.74	61.42±15.43	0.7573	0.03
BMI (kg/m^2^), n (%)	<18.5	28 (6.54)	15 (7.11)	13 (5.99)	0.4408	0.01	53 (6.31)	26 (6.17)	27 (6.46)	0.8220	<0.01
	18.5–24	251 (58.64)	126 (59.72)	125 (57.60)		0.02	498 (59.53)	254 (59.74)	245 (59.31)		<0.01
	≥24	149 (34.81)	70 (33.18)	79 (36.41)		0.03	286 (34.16)	145 (34.09)	141 (34.23)		<0.01
ASA score, *n* (%)	1	13 (3.04)	6 (2.84)	7 (3.23)	0.2901	<0.01	22 (2.64)	11 (2.53)	11 (2.75)	0.7939	<0.01
	2	372 (86.92)	188 (89.10)	184 (84.79)		0.04	729 (87.01)	373 (87.86)	356 (86.13)		0.02
	3	43 (10.05)	17 (8.06)	26 (11.98)		0.04	87 (10.36)	41 (9.60)	46 (11.13)		0.02
Blood loss (ml), median (IQR)		400.00 (300.00–700.00)	400.00 (300.00–800.00)	300.00 (200.00–600.00)	0.0123	0.12	400.00 (250.00–650.00)	400.00 (250.00–800.00)	300.00 (200.00–600.00)	0.0271	0.08
NRS2002 score, *n* (%)	<3	333 (77.80)	173 (81.99)	160 (73.73)	0.0399	0.08	644 (76.89)	332 (78.28)	312 (75.45)	0.3314	0.03
	≥3	95 (22.20)	38 (18.01)	57 (26.27)			194 (23.11)	92 (21.72)	101 (24.55)		
Preoperative biliary drainage, *n* (%)		69 (16.12)	38 (18.01)	31 (14.29)	0.2949	0.04	141 (16.80)	77 (18.24)	63 (15.33)	0.2599	0.03
Preoperative chemotherapy, *n* (%)		43 (10.05)	36 (17.06)	7 (3.23)	<0.0001	0.14	64 (7.70)	42 (9.92)	22 (5.41)	0.0143	0.05
Preoperative radiotherapy, *n* (%)		26 (6.07)	19 (9.00)	7 (3.23)	0.0123	0.06	47 (5.67)	25 (5.92)	22 (5.41)	0.7498	0.01
History of hypertension, *n* (%)		142 (33.18)	78 (36.97)	64 (29.49)	0.1006	0.07	276 (32.90)	141 (33.12)	135 (32.68)	0.8909	<0.01
History of diabetes, *n* (%)		104 (24.30)	61 (28.91)	43 (19.82)	0.0283	0.09	219 (26.14)	109 (25.71)	110 (26.58)	0.7745	0.01
History of pancreatitis, *n* (%)		38 (8.88)	18 (8.53)	20 (9.22)	0.8031	0.01	71 (8.44)	35 (8.31)	35 (8.59)	0.8838	<0.01
History of abdominal operation, *n* (%)		122 (28.50)	60 (28.44)	62 (28.57)	0.9753	<0.01	240 (28.66)	121 (28.51)	119 (28.82)	0.9226	<0.01
Operative time (minute), median (IQR)		180.00 (170.00–215.00)	180.00 (155.00–210.00)	190.00 (170.00–220.00)	0.0078	0.22	180.00 (170.00–220.00)	180.00 (170.00–230.00)	185.00 (170.00–215.00)	0.5485	0.01
Surgical method, *n* (%)	PD	415 (96.96)	209 (99.05)	206 (94.93)	0.0130	0.04	815 (97.32)	415 (97.77)	400 (96.85)	0.4117	0.01
	PPPD	13 (3.04)	2 (0.95)	11 (5.07)			22 (2.68)	9 (2.23)	13 (3.15)		
Vascular resection, *n* (%)		36 (8.41)	19 (9.00)	17 (7.83)	0.6627	0.01	60 (7.16)	29 (6.90)	31 (7.42)	0.7681	0.01
Pancreatic texture, *n* (%)	Soft	199 (46.50)	81 (38.39)	118 (54.38)	<0.0001	0.16	386 (46.14)	194 (45.59)	193 (46.72)	0.7878	0.01
	Median	190 (44.39)	99 (46.92)	91 (41.94)		0.05	373 (44.54)	191 (45.05)	182 (44.02)		0.01
	Hard	39 (9.11)	31 (14.69)	8 (3.69)		0.11	78 (9.32)	40 (9.37)	38 (9.27)		<0.01
Main pancreatic duct, *n* (%)	<2 mm	62 (14.49)	30 (14.22)	32 (14.75)	0.0002	0.01	121 (14.43)	61 (14.43)	60 (14.42)	0.5148	<0.01
	2–5 mm	247 (57.71)	100 (47.39)	147 (67.74)		0.20	475 (56.71)	240 (56.45)	235 (56.99)		0.01
	>5 mm	119 (27.80)	81 (38.39)	38 (17.51)		0.21	242 (28.86)	124 (29.12)	118 (28.59)		0.01
Intraoperative blood transfusion, *n* (%)		25 (5.84)	12 (5.69)	13 (5.99)	0.8935	<0.01	46 (5.53)	21 (4.83)	26 (6.24)	0.3724	0.01
Preoperative total bilirubin, median (IQR)		17.45 (10.90–112.10)	15.80 (9.60–80.90)	18.60 (12.50–129.30)	0.0145	0.12	17.20 (10.60–96.70)	17.20 (9.90–88.70)	16.90 (11.20–108.90)	0.3255	0.04
Preoperative albumin, mean±SD		41.92±4.26	41.86±4.17	41.98±4.36	0.7818	0.03	42.23±5.83	42.26±5.93	42.21±5.74	0.9054	0.01
Preoperative prealbumin, mean±SD		208.82±59.29	206.20±58.48	211.35±60.09	0.3695	0.09	209.91±79.27	209.70±80.88	210.13±77.87	0.9384	0.01
Preoperative haemoglobin, mean±SD		129.55±19.11	127.31±19.88	131.72±18.12	0.0169	0.23	130.56±25.96	130.20±27.91	130.92±23.96	0.6912	0.04
Pathology, *n* (%)	Malignant	313 (73.13)	157 (74.41)	156 (71.89)	0.5568	0.03	604 (72.17)	306 (71.98)	299 (72.35)	0.9049	<0.01
	Benign	115 (26.87)	54 (25.59)	61 (28.11)			233 (27.83)	119 (28.02)	114 (27.65)		

Cause of the weight of observation after PS weighting may not be an integer, the count of each group may be less or more than the total of all groups after rounding off.

ASA, American Society of Anesthesiologists; EOF, early oral feeding; IQR, interquartile range; NJEEN, nasojejunal early enteral nutrition; PD, pancreaticoduodenectomy; PPPD, pylorus-preserving pancreaticoduodenectomy; PS, propensity score.

### Operative outcomes

**Figure 3:**
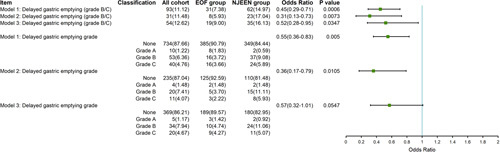
The odd ratios (95% CI) for delayed gastric emptying grade. EOF, early oral feeding; NJEEN, nasojejunal early enteral nutrition.

The crude incidence of DGE (B/C) before PSW methods was 9.00% (19/211) in the EOF group and 16.13% (35/217) in the NJEEN group. After PSW, the overall rate of clinically relevant DGE (B/C) was 11.12% (93/837). It was found that patients who underwent EOF experienced a significantly lower DGE (B/C) rate (Table 2) than patients who underwent NJEEN [7.38% (31/424) vs. 14.97% (62/413), *P*=0.0005, odds ratio= 0.45, 95% CI = 0.29–0.71, *P* = 0.0006, Figure 3]. The sensitivity analyses yielded consistent results. The overall DGE grade (Z=2.30, *P*=0.0215, odds ratio= 0.55, 95% CI = 0.36–0.83, *P* = 0.0050, Fig. 3) and the rate of Clavien–Dindo Grade III or higher [6.36% (27/424) vs. 12.29% (51/413), *P*=0.0031], and surgical site infection [20.11% (85/424) vs. 29.31% (121/413), *P*=0.0020] in the EOF group were significantly lower than the NJEEN group. These findings were consistent with the results of one sensitivity analysis (Table 2). The EOF group was associated with a lower rate of pancreatic fistulas (grade B/C) [9.20% (39/424) vs. 14.68% (61/413), *P*=0.0144], as well as a lower rate of postoperative gastrointestinal endoscopic interventions [3.19% (14/424) vs. 7.88% (33/413), *P*=0.0029]. However, the sensitivity analysis did not yield the same results (Table 2). In all three models, no significant differences in other outcomes were found between both groups.

**Table 2:** Relationship between nutritional approaches and outcomes of patients’ postoperative conditions.

Variables	Classification	EOF group after PSW (*N*=424)	NJEEN group after PSW (*N*=413)	Statistic	*P*
Delayed gastric emptying grade, n (%)	None	385 (90.79)	349 (84.44)	2.30	0.0215
	Grade A	8 (1.83)	2 (0.59)		
	Grade B	16 (3.72)	37 (9.08)		
	Grade C	16 (3.66)	24 (5.89)		
Delayed gastric emptying (grade B/C), *n* (%)		31 (7.38)	62 (14.97)	12.18	0.0005
Pancreatic fistula (grade B/C), *n* (%)		39 (9.20)	61 (14.68)	5.99	0.0144
Chyle leak, *n* (%)		9 (2.15)	17 (4.10)	2.64	0.1044
Pneumonia infections, *n* (%)		5 (1.24)	2 (0.39)	1.85	0.1736
Surgical site infection, *n* (%)		85 (20.11)	121 (29.31)	9.53	0.0020
Postoperative haemorrhage, n (%)		17 (4.01)	14 (3.32)	0.28	0.5960
Acute pancreatitis, *n* (%)		0	4 (0.87)	3.69	0.0547
TPN use, *n* (%)		57 (13.38)	70 (17.02)	2.15	0.1422
Postoperative gastrointestinal endoscopic intervention, *n* (%)		14 (3.19)	33 (7.88)	8.85	0.0029
DSA, *n* (%)		3 (0.61)	5 (1.12)	0.65	0.4208
Clavien–Dindo Grades, *n* (%)	Grade I	238 (56.12)	242 (58.50)	−0.20	0.8425
	Grade II	159 (37.52)	121 (29.20)		
	Grade IIIa	18 (4.18)	41 (9.82)		
	Grade IIIb	3 (0.63)	0		
	Grade IVa	3 (0.77)	7 (1.70)		
	Grade IVb	0	0		
	Grade V	3 (0.79)	3 (0.76)		
Clavien–Dindo Grade III or higher, *n* (%)		27 (6.36)	51 (12.29)	8.73	0.0031
30-day mortality, *n* (%)		3 (0.79)	3 (0.76)	0.00	0.9701
Postoperative LOS, median (IQR)		9.00 (7.00–13.00)	10.00 (7.00–14.00)	1.00	0.3175
30-day readmission, *n* (%)		18 (4.24)	21 (5.09)	0.34	0.5601
Cost (dollars), mean±SD		9691.99±6790.73	9967.98±5823.49	−0.63	0.5278

Cause of the weight of observation after PS weighting may not be an integer, the count of each group may be less or more than the total of all groups after rounding off.

DSA, digital subtraction angiography; EOF, early oral feeding; IQR, interquartile range; LOS, length of stay; NJEEN, nasojejunal early enteral nutrition; PSW, propensity score weighting; TPN, total parenteral nutrition.

Table 2 presents the 30-day mortality rates and other outcomes. There was no difference in 30-day mortality rates [0.79% (3/424) vs. 0.76% (3/413), *P*=0.9701], postoperative length of stay [9.00 (7.00–13.00) vs. 10.00 (7.00–14.00), *P*=0.3175], 30-day readmission [4.24% (18/424) vs. 5.09% (21/413), *P*=0.5601], and costs (9691.99±6790.73 vs. 9967.98±5823.49, *P*=0.5278) between the two groups. Details of operative outcomes on sensitivity analyses are provided in the Supplementary Appendix, Supplemental Digital Content 1, http://links.lww.com/JS9/B84.

### Subgroup analyses

Subgroup analyses according to the presence of a soft pancreatic texture yielded consistent results (Table 3). The EOF group was associated with lower DGE grade (Z=4.22, *P*<0.0001) and rate of DGE (B/C) [5.90% (11/194) vs. 22.07% (43/193), *P*<0.0001], postoperative gastrointestinal endoscopic intervention [1.16% (2/194) vs. 10.36% (20/193), *P*=0.0001], and Clavien–Dindo Grade III or higher [1.97% (4/194) vs. 15.26% (29/193), *P*<0.0001]. Both sensitivity analyses (Table 3) yielded similar conclusions. The main analysis and one sensitivity analysis revealed that the EOF group was associated with a significantly lower rate of surgical site infection [22.92% (44/194) vs. 32.01% (62/193), *P*=0.0452] and total parenteral nutrition use [14.61% (28/194) vs. 24.05% (46/193), *P*=0.0188] than the NJEEN group (Table 3). The EOF group was associated with a lower risk of 30-day readmission [3.42% (7/194) vs. 8.96% (17/193), *P*=0.0239], although sensitivity analyses did not support these findings. Details of subgroup analyses on sensitivity analyses are provided in the Supplementary Appendix, Supplemental Digital Content 1, http://links.lww.com/JS9/B84.

**Table 3:** Relationship between nutritional approaches and outcomes of patients’ postoperative conditions of patients with soft pancreatic texture.

Variables	Classification	EOF group after PSW (*N*=194)	NJEEN group after PSW (*N*=193)	Statistic	*P*
Delayed gastric emptying grade, *n* (%)	None	182 (94.10)	148 (76.67)	4.22	<0.0001
	Grade A	0	2 (1.26)		
	Grade B	7 (3.72)	30 (15.38)		
	Grade C	4 (2.17)	13 (6.69)		
Delayed gastric emptying (grade B/C), *n* (%)		11 (5.90)	43 (22.07)	21.03	<0.0001
Pancreatic fistula (grade B/C), *n* (%)		28 (14.71)	39 (20.29)	2.09	0.1487
Chyle leak, *n* (%)		9 (4.71)	12 (5.98)	0.31	0.5804
Pneumonia infections, *n* (%)		3 (1.33)	0	2.59	0.1077
Surgical site infection, *n* (%)		44 (22.92)	62 (32.01)	4.01	0.0452
Postoperative haemorrhage, *n* (%)		6 (3.02)	9 (4.54)	0.61	0.4331
Acute pancreatitis, *n* (%)		0	4 (1.85)	3.62	0.0571
TPN use, *n* (%)		28 (14.61)	46 (24.05)	5.52	0.0188
Postoperative gastrointestinal endoscopic intervention, *n* (%)		2 (1.16)	20 (10.36)	15.10	0.0001
DSA, *n* (%)		2 (0.82)	5 (2.40)	1.53	0.2154
Clavien–Dindo Grades, *n* (%)	Grade I	96 (49.42)	94 (48.57)	0.86	0.3907
	Grade II	94 (48.61)	70 (36.17)		
	Grade IIIa	2 (1.16)	28 (14.52)		
	Grade IIIb	0	0		
	Grade IVa	2 (0.82)	1 (0.75)		
	Grade IVb	0	0		
	Grade V	0	0		
Clavien–Dindo Grade III or higher, *n* (%)		4 (1.97)	29 (15.26)	21.69	<0.0001
30-day mortality, *n* (%)		0	0	—	—
Postoperative LOS, median (IQR)		10.00 (8.00–14.00)	11.00 (8.00–15.00)	0.62	0.5323
30-day readmission, *n* (%)		7 (3.42)	17 (8.96)	5.11	0.0239
Cost (dollars), mean±SD		9556.46±6780.58	10182.6±5965.3	−0.93	0.3538

Cause of the weight of observation after PS weighting may not be an integer, the count of each group may be less or more than the total of all groups after rounding off.

DSA, digital subtraction angiography; EOF, early oral feeding; IQR, interquartile range; LOS, length of stay; NJEEN, nasojejunal early enteral nutrition; PSW, propensity score weighting; TPN, total parenteral nutrition.

## Discussion

To our knowledge, our study represents the largest cohort comparing EOF with NJEEN after PD. Previous studies supporting EOF after PD had smaller sample sizes, and their conclusions were often based on the pooled effects of multiple measures in the ERAS program^19,20^. Herein, we sought to assess the impact of EOF on patients that underwent PD. We revealed that EOF following PD is a safe and feasible practice associated with a lower DGE grade B/C rate.

Over the past few years, the ERAS concept has become increasingly popular in physical therapy. According to the ERAS guidelines, a normal diet is recommended after PD^21^. However, Bozzetti *et al.* ^22^ reported that patients with pancreatic cancer were more likely to suffer from malnutrition, and perioperative nutritional support for EEN should be considered in addition to ESPEN recommendations. The authors advocated that EOF is not always feasible after PD. Nonetheless, two meta-analyses revealed that EEN after PD was safe and effective, did not increase the risk of complications, and could shorten the length of hospital stay^9,10^. As a result, EEN after PD is still commonly recommended in clinical practice.

Previous studies have yielded inconsistent findings, with some endorsing EEN and others favoring EOF. In a study by Gerritsen *et al.* ^19^, EOF reduced the time to resumption of adequate oral intake and length of hospital stay after PD without adversely affecting postoperative morbidity. However, this study included a relatively small sample size, which did not provide the necessary power to validate the superiority of EOF. Some studies have focused exclusively on patients that underwent PPPD^14^. The findings of our study provide compelling evidence regarding the impact of EOF after PD.

Our study findings contrast with reports in the literature^23^. Rayar *et al.* documented that EN reduces DGE following PD. It should be noted, however, that in their study, the NGT was kept until at least POD5, whereas in our study, the NGT was removed when the gastric juice volume was less than 300 ml per 24 h on POD 2 after surgery. Based on our experience, early extubation of NJT and EOF may be more beneficial in promoting the recovery of gastrointestinal function. The present study revealed an overall incidence of grade B and C DGE of 12.62%, significantly lower than that reported by Rayar and colleagues.

Bárdos *et al.* ^24^ found that distention and satiety decreased fluid intake in rats depending on intensity. According to Liu *et al.* ^14^, intestinal contents may also be responsible for DGE following PPPD. Importantly, the present study provides hitherto undocumented evidence that NJEEN is associated with higher grade B and C DGE incidence than EOF.

Currently, there are no relevant studies related to nutritional support following PD in high-risk cases of POPF, such as those with a soft pancreatic texture. As per the ISGPS Guidelines, the placement of a feeding tube is recommended for patients at high risk of POPF, based on a Fistula Risk Score greater than or equal to 7^15^. Nonetheless, this recommendation was based on grade C evidence. It is now understood that high-risk factors for POPF include a soft gland texture, a small pancreatic duct diameter, intraoperative blood loss, and specific disease pathologies^25^.

As part of our study, a subgroup analysis assessed whether patients with a soft pancreatic texture were at high risk for POPF after PD. Our results revealed that the EOF group was associated with a lower incidence of total DGE, grade B/C DGE, Clavien–Dindo grade 3 and above complications, and fewer endoscopic interventions than the NJEEN group. Importantly, our results suggest that NJEEN should not be not routinely recommended for patients with high-risk conditions, especially those with soft pancreatic texture after PD. Accordingly, our findings do not align with the recommendation provided by the ISGPS.

Perinel *et al.* ^12^ reported that NJEEN was associated with an increased incidence of total complications after PD and a higher incidence and severity of POPF. Consistently, in this study, there was a higher incidence of pancreatic fistula and surgical site infection in the NJEEN group following PD than in the EOF group following PSW. Accordingly, NJEEN may increase the risk of pancreatic fistulas and surgical site infections following PD, leading to an increased risk of DGE and other complications.

Amenu *et al.* ^26^ described a rare enteral nutrition complication involving bowel ischaemia, intestinal pneumatosis, and hepatic portal venous gas following Whipple’s procedure for pancreatic cancer. According to their findings, enteral nutrition administered during ileus may lead to bacterial overgrowth, resulting in progressive distension, and generate intraluminal toxins that directly damage the mucosa^27^. Indeed, excessive and rapid enteral nutrition might cause distension and bacterial translocation, potentially leading to delayed gastric emptying and infection at the surgical site.

Some limitations of this study should be acknowledged. Firstly, the study design is retrospective and focused on a single group, which may limit the generalizability of our findings. Although this design ensures optimal baseline data consistency, it cannot establish causation or fully control for potential confounding factors. Therefore, a prospective study is warranted to validate and further explore the potential benefits of early oral feeding strategies following PD.

## Conclusion

Following PD, early oral feeding demonstrates several advantages over NJEEN as it effectively reduces the incidence of grade B/C DGE. Moreover, the EOF procedure is proven to be safe and feasible, making it a compelling choice for postoperative feeding management following PD. As a result, it is recommended to consider EOF as the preferred approach in these cases.

## Ethical approval

The studies involving human participants were reviewed and approved by Institutional Review Board of Changhai Hospital(No.CHEC2022-136). And this study has been submitted to the Clinical Trials Register (NCT05573399).

## Source of funding

The research was funded by resources from the Shanghai ShenKang hospital development centre (No. SHDC2020CR2001A), the Pilot Program of Naval Medical University (Cheng WU), and the First Affiliated Hospital of Navy Military Medical University (No. 2020YXK006).

## Author contribution

W.J., S.W., S.G., and X.S. contributed equally to this work. W.J., S.W., S.G., and X.S. had full access to all of the data in the study and take responsibility for the integrity of the data and the accuracy of the data analysis. Study design: W.J., C.W., and G.J. Data collection: W.J., S.G., W.L., X.S., Y.R., L.O., K.Z., and S.G. Drafting of the manuscript: W.J., S.W., and S.G. Administrative, technical, or material support: C.W. Critical revision of the manuscript: Gang Jin.

## Conflicts of interest disclosure

The authors declare no conflicts of interest. The research was funded by resources from the Shanghai ShenKang hospital development centre (No. SHDC2020CR2001A), the Pilot Program of Naval Medical University (Cheng WU), and the First Affiliated Hospital of Navy Military Medical University (No. 2020YXK006). Data were recorded and stored in our prospective database, in conjunction with local data management standard operating procedures. Anonymized data were collated centrally at the chief investigator’s institution. All patients were service users of the state funded point-of-access care provided by the surgical units included, and only routinely available audited data were collected. As such, the collection of the data used in this study was deemed a service evaluation audit which did not require ethical approval, only approval from individual organisations participating in the service evaluation was required and granted.

## Research registration unique identifying number (UIN)

1. Name of the registry:Early Oral Feeding for Patients After Pancreaticoduodenectomy.
2. Unique Identifying number or registration ID:NCT05573399.
3. Hyperlink to your specific registration : https://clinicaltrials.gov/ct2/show/NCT05573399?term=NCT05573399&draw=2&rank=1.

## Guarantor

Professor Cheng Wu. E-mail: wucheng@smmu.edu.cn Professor Gang Jin (principal investigator) E-mail: jingang@smmu.edu.cn
